# The use of isotope injections in sentinel node biopsy for breast cancer: are the 1- and 2-day protocols equally effective?

**DOI:** 10.1186/s40064-015-1314-y

**Published:** 2015-09-15

**Authors:** Nazera Dodia, Deena El-Sharief, Cliona C. Kirwan

**Affiliations:** Manchester Medical School, University of Manchester, Stopford Building, Oxford Rd, Manchester, M13 9PT UK; Department of Breast Surgery, University Hospital of South Manchester, Southmoor Road, Wythenshawe, Manchester, M23 9LT UK; Institute of Cancer Sciences, University of Manchester, University Hospital of South Manchester, Southmoor Road, Wythenshawe, Manchester, M23 9LT UK

**Keywords:** Sentinel lymph node biopsy, Breast cancer, Isotope, Surgery

## Abstract

Sentinel lymph nodes are mapped using ^99m^Technetium, injected on day of surgery (1-day protocol) or day before (2-day protocol). This retrospective cohort study compares efficacy between the two protocols. Histopathology for all unilateral sentinel lymph node biopsies (March 2012–March 2013) in a single centre were reviewed. Number of sentinel lymph nodes, non-sentinel lymph nodes and pathology was compared. 2/270 (0.7 %) in 1-day protocol and 8/192 (4 %) in 2-day protocol had no sentinel lymph nodes removed (p = 0.02). The median (range) number of sentinel lymph nodes removed per patient was 2 (0–7) and 1 (0–11) in the 1- and 2-day protocols respectively (p = 0.08). There was a trend for removing more non-sentinel lymph nodes in 2-day protocol [1-day: 52/270 (19 %); 2-day: 50/192 (26 %), p = 0.07]. Using 2-day, sentinel lymph node identification failure rate is higher, although within acceptable rates. The 1 and 2 day protocols are both effective, therefore choice of protocol should be driven by patient convenience and hospital efficiency. However, this study raises the possibility that 1-day may be preferable when higher sentinel lymph node count is beneficial, for example following neoadjuvant chemotherapy.

## Background

Over the last decade sentinel lymph node biopsy (SLNB) has become the accepted method for staging of the axilla in patients with breast cancer and clinically node negative disease (Giuliano et al. 1994; Veronesi et al. 2003; Mansel et al. 2006).

Although early trials highlight an improved accuracy of SLNB with the removal of multiple nodes (Wong et al. 2001), current evidence shows that in low risk tumours, the dissection of fewer SLNs still achieves an accurate biopsy result (Low and Littlejohn 2006). It is important to establish the optimal number of nodes to be biopsied as an over-dissection can lead to an increased risk of lymphoedema, sensory deficit and impaired shoulder mobility (Mansel et al. 2006). In specific cases, taking more SLNs can be beneficial for example, there is evidence that larger numbers of SLNs reduce the risk of a false negative biopsy result in patients who have undergone neoadjuvant chemotherapy (Boughey et al. 2013; Kuehn et al. 2013).

In the UK, current NICE guidelines (NICE 2009) recommend performing SLNB using the dual technique with isotope and blue dye. Of screen detected invasive breast cancers, 84 % undergo SLNB in the UK (West Midlands NHS Breast Screening Quality assurance Centre 2013).

At our hospital, all women diagnosed with breast cancer and clinically node negative disease (based on axillary ultrasound findings ± cytology or core biopsy) undergo SLNB. Lymphatic mapping is carried out using the dual technique of ^99m^Technetium isotope and patent blue V.

To increase efficiency the unit has two protocols for SLNB based on when the ^99m^Technetium isotope is injected pre-operatively: the 1-day protocol where the isotope is injected on the day of surgery; and the 2-day protocol where patients are injected the day before. The 1-day protocol is allocated to patients who undergo surgery on the first day of a working week, patients who require wire guided tumour localisation prior to surgery or patients who are listed towards the end of the working day. All other patients are usually allocated the 2-day protocol. Previous authors have adopted similar protocols (Winchester et al. 1999; McCarter et al. 2001; Yeung et al. 2001). The 2-day protocol prevents delays to morning lists in a hospital where the isotope is administered in a department geographically separate from the operating theatres and thus increases productivity. Thus these protocols are partly driven by the pragmatics of efficiency for patients and the hospital. To minimise the loss of signal from the isotope an approximately double dose is used in the 2-day protocol, however anecdotally surgeons have reported reduced isotope signal using the 2-day protocol compared to the 1-day protocol.

We hypothesised that (as a result of weakened signal, particularly in secondary and tertiary SLNs) fewer SLNs and more NSLNs would be removed using the 2-day vs 1-day protocol. We aimed to examine whether the any difference in the efficacy of these two techniques could have a clinical impact.

## Patients and methods

This was a single centre, retrospective cohort study of all women with unilateral breast cancer, diagnosed between March 2012 and March 2013, with clinically node negative disease at presentation who required SLNB.

### Sentinel lymph node mapping and biopsy technique

The isotope is prepared off-site and delivered daily to the hospital at 7am and 10.30am. Experienced radiographers administer the isotope intra-dermally into the areola, in the quadrant of the tumour. In the 1-day protocol 20 MBq of 0.1–0.2 ml ^99m^Technetium-labeled sulphur colloid is injected at approximately 9am on the morning of the surgery; in the 2-day protocol 40 MBq of 0.1–0.2 ml ^99m^Technetium-labeled sulphur colloid is injected at approximately 3 pm the day before surgery. The isotope injection site is localised to the edge of the areola, in the upper outer quadrant of the breast. In addition, 2 ml of 2.5 % patent blue dye is injected after the patient is anaesthetised. The injection is given sub-dermally at the edge of the areola in the upper outer quadrant or in the quadrant of the tumour, depending on the preference of the surgeon. Lymphoscintigrams are not used. Nodes that show radioactivity, using the gamma probe (‘hot’ nodes), are removed and labelled as SLNs. In addition nodes that are stained blue (with or without radioactivity) are also labelled SLNs. Any non-sentinel lymph nodes (NSLN) removed (e.g. found to contain no signal following removal and no blue staining, or removed as part of a sample in the instance of mapping failure) are labelled as ‘non-sentinel nodes’. We aim to remove a maximum of four SLNs, however clinically suspicious nodes may be removed at the surgeon’s discretion. All nodes are sent for histological analysis.

### Data collection and analysis

In all patients, the protocol (1- or 2-day), isotope dose, number of SLNs and NSLNs removed, the total number of nodes removed and nodal histology were recorded.

Data was analysed using SPSS version 15. Chi squared, Fisher’s exact, Mann–Whitney U and Spearman’s correlation were used to analyse the data.

## Results

Of 462 patients undergoing SLNB in the 12-month study period, 270 had SLNB using the 1-day protocol and 192 using the 2-day protocol. The mean dose of isotope used was 22.3 [confidence interval (CI) 22.0–22.7 MBq] MBq for the 1-day protocol and 40.4 [CI (39.9–40.9)] MBq for the 2-day protocol.

Ten patients had a failed sentinel node biopsy (no sentinel node and only non-sentinel nodes removed), with 2/270 (0.7 %) in the 1-day protocol and 8/192 (4 %) in the 2-day protocol (p = 0.02). Of these ten patients, only one patient had lymph node metastases (2 of 5 nodes positive, 2-day protocol).

The median total number of nodes per patient was similar between the 1 and 2-day protocols [1-day: 2 (0–7), 2-day: 2 (0–11), p = 0.7], however there appeared to be a greater number of sentinel nodes removed on the 1-day protocol and non-sentinel nodes on the 2-day protocol.

**Fig. 1:**
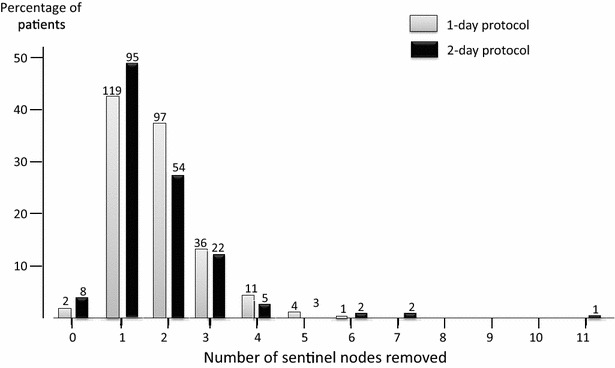
Percentage of patients with different numbers of sentinel lymph nodes (SLNs) removed at sentinel lymph node biopsy, separated into 1-day (*grey*, n = 270) and 2-day (*black*, n = 192 protocols). The median (range) number of SLNs removed per patient was 2 (0–6) in the 1-day protocol and 1 (0–11) for the 2-day protocol (p = 0.08). There was a trend for a higher percentage of patients having two or more SNs removed with the 1-day protocol (55 %) compared with the 2-day protocol (46 %) (p = 0.06). Bar chart is shown as percentage of patients, with absolute *numbers given above individual bars*

The median (range) number of SLNs removed per patient was 2 (0–6) in the 1-day protocol and 1 (range 0–11) for the 2-day protocol (p = 0.08) (Fig. 1).

**Fig. 2:**
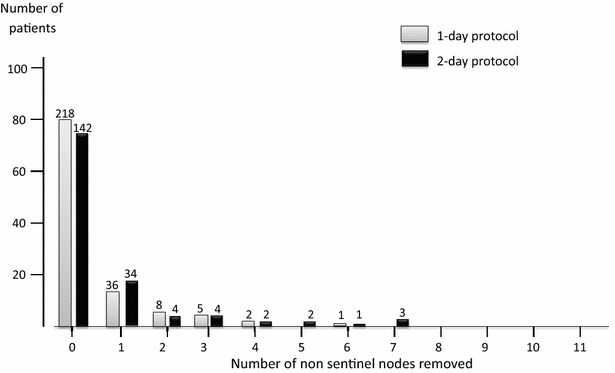
Percentage of patients with different numbers of non-sentinel lymph nodes (NSLNs) removed at sentinel lymph node biopsy, separated into 1-day (*grey*, n = 270) and 2-day (*black*, n = 192) protocols. There was a trend for a higher proportion of patients having one or more NSLN removed in the 2-day protocol (1-day: 52/270 (19 %); 2-day: 50/192 (26 %), p = 0.07). Bar chart is shown as percentage of patients, with absolute *numbers given above individual bars*

There was a trend for a higher proportion of patients having one or more NSLNs removed in the 2-day [50/192 (26 %) than the 1-day protocol (52/270 [19 %]) (p = 0.07) (Fig. 2).

As expected, there was an inverse correlation between number of SLNs and number of NSLNs removed (p = 0.002, Spearman’s correlation −0.12).

Lymph node metastases were found in 15 patients (5.5 %; 13 macrometastases, 2 micrometastases) on the 1-day protocol and 16 patients (8.3 %; 13 macrometastases, 3 micrometastases) on the 2-day protocol. Only one patient had a micrometastasis in a NSLN in the presence of normal SLNs, indicating a falsely negative SLNB, however no further disease was found on completion axillary node clearance. One patient had two macrometastases in NSLNs, with extracapsular spread, without any SLNs being removed, implying a failure of lymphatic mapping, however this was recognised at surgery with the histological sample labelled ‘axillary sample’.

## Discussion

This study compares the 1- and 2-day protocols for sentinel node biopsy. It aimed to determine whether there was any clinically relevant difference in the two techniques, where choice of technique is largely driven by pragmatics and efficiency.

There is an increased rate of failure of sentinel node technique using the 2-day protocol compared with the 1-day protocol. In published literature, the failure rate of identifying the sentinel node is 2–8 % (Veronesi et al. 1997; Giuliano et al. 1997; Albertini et al. 1996; Krag et al. 1998). Using the combined blue dye and isotope techniques, UK and American guidelines for surgeons new to SLNB recommend a maximum rate of 10 and 15 % false negative respectively, before using the SLNB technique independently (Association of Breast Surgery at B 2009; Simmons 2001). Although the 2-day protocol failure rate in our study is statistically significantly higher, whether this is clinically significant is questionable given that at 4 %, the failure rate is still within the clinically acceptable level.

Surgeons anecdotally report that 1-day protocols can result in a relatively ‘noisy’ axilla. We hypothesised that secondary and tertiary sentinel nodes may have a detectable signal with the ‘noisy’ one-day protocol, but become undetectable with the less ‘noisy’ 2-day protocol.

This is supported by our finding of a trend for greater number of SLNs being removed with the 1-day protocol. It is possible that surgeons were removing many hot nodes before finding the hottest node. The NEW START programme defines SLNs as any nodes with >10 % of the count of the node with the maximum count rate. In this current study we do not have the count rates of the SLNs removed so cannot verify if they are true SLNs as defined by NEW START (MacNeill et al. 2005). In addition, some of our SLNs may have been ‘blue’ and not ‘hot’, however it is recognised that only 5 % of identified SLNs are ‘blue’ and not ‘hot’, so this is unlikely to have a significant impact on our findings (Krag et al. 2007).

Our study provides a potential argument for using a 1-day protocol in selected patients where the sensitivity of SLNB is questioned and may be improved by a higher yield of SLNs, for example, following neoadjuvant chemotherapy (Boughey et al. 2013; Kuehn et al. 2013). In patients receiving neoadjuvant chemotherapy for clinically node negative disease, some authors report a higher false negative rate of SLNB when performed after chemotherapy than prior to chemotherapy (Papa et al. 2008; Kang et al. 2004). In biopsy proven node positive patients undergoing SLNB following neoadjuvant chemotherapy in the ACOSOG Z1071 Trial, the false negative rate was 10 % or less as long as a minimum of 2 SLNs were removed (Boughey et al. 2013). In our current study, there was a trend for a higher percentage of patients having two or more SLNs removed with the 1-day protocol (55 %) compared with the 2-day protocol (46 %) (p = 0.06), highlighting that the 1-day protocol may be beneficial in this subset of patients, through a possible increase in SLN yield, however this is clearly hypothesis generating.

Unlike previous studies, here we also report rates of NSLNs removed. The high percentage of NSLNs taken with the 2-day protocol may reflect difficulty in identifying the SLN. Anecdotally surgeons report ‘less noise’ in the axilla in the 2-day protocol, suggesting that there is greater signal fade with the 2-day protocol, despite a double dose of isotope. This would result in a quieter signal in SLNs, making them harder to find. This hypothesis is supported by our current data, with a trend for higher number of SLNs removed with the 1-day protocol compared with the 2-day protocol and more NSLNs found in the 2-day protocol. With increased difficulty in locating SLNs (due to weaker signal) the surgeon may take NSLNs anatomically close to the SLN by mistake if they are easily palpable, or have to resort to an axillary node sample. This is further supported by the inverse correlation between number of sentinel nodes and non-sentinel nodes removed. It is interesting that previous studies comparing 1- and 2-day protocols have not commented on rates of NSLN removal. Our findings are at odds with McCarter et al., who found that more SLNs were found on lymphoscintigraphy on the 2-day protocol (McCarter et al. 2001). It is likely that their study contradicts ours because the isotope dose was increased by five between their 1- and 2-day protocols, which may further support the hypothesis above that protocol technique can be adapted to increase the SLN yield in situations where this is beneficial (e.g. post neoadjuvant chemotherapy). In our current study, despite ^99m^Technetium having a relatively short half-life of only 6 h, and a relatively smaller dose used compared to the previous study, the 2-day signal was sufficient for successful SLNB in 96 % of patients.

In this current series, removal of NSLNs added no additional histological information in all but two cases (of which one was a recognised failed SLNB). Removing more NSLNs may suggest a less directed SLNB has been performed, which could potentially increase the risk of morbidity, through additional axillary dissection.

It needs to be acknowledged that in this current cohort study, 1-day patients are a different clinical group to 2-day patients. The 1-day protocol is largely used for patients who need wire localization of a small lesion, whereas 2-day patients usually require ultrasound localisation of a mass lesion, or have clinically palpable lesions requiring no localisation or are undergoing mastectomy. This retrospective study therefore has a clear selection bias with 2-day patients tending to have larger tumours and more advanced disease. The difference in failure rate may possibly be related to axillary disease as well as technical failure, although node-positivity rate was not significantly higher in the 2-day protocol, despite the presumed selection bias. It is noteworthy that only one of the ten failed SLNB patients had metastases in their NSLNs. It is possible, however, that more NSLNs could be deliberately biopsied in the 2-day protocol due to more clinical concern.

A limitation of this study is that our data did not include details of time from injection to surgery. Potentially some patients on the 1-day protocol could have had surgery up to 8 h after injection, and patients on the 2-day protocol, had surgery only 16 h after injection, blurring the boundaries between these two groups.

## Conclusions

This study highlights that the 2-day protocol has a higher failure rate and may make identifying SLNs more challenging, with resultant increased NSLN removal, and therefore potentially greater axillary dissection. However the true clinical significance of this is likely to be small. Here we have demonstrated that both the 1- and 2-day protocols are clinically safe and largely equivalent. Choice of protocol should be driven by patient convenience and hospital efficiency. However, considering the potentially higher SLN yield achieved with the 1-day protocol, we hypothesise that the 1-day protocol is preferential in the subset of patients where a high SLN yield may influence clinical management, for example following neoadjuvant chemotherapy.

## Abbreviations

SLNB: sentinel lymph node biopsy
SLN: sentinel lymph node
NSLN: non-sentinel lymph node
